# Mindfulness mediates the association between chronotype and depressive symptoms in young adults

**DOI:** 10.1371/journal.pone.0319915

**Published:** 2025-03-19

**Authors:** Gulin Yatagan Sevim, Tina Yuet Law, Simon L. Evans

**Affiliations:** Faculty of Health and Medical Sciences, School of Psychology, University of Surrey, Guildford, Surrey, United Kingdom; Nofer Institute of Occupational Medicine, POLAND

## Abstract

**Background:**

Chronotype influences risk of depression, with evening-types at higher risk, although the reasons for this are uncertain. Potential mediating factors include mindfulness, sleep quality, rumination, and alcohol consumption, but research is lacking.

**Methods:**

We explored the role of these factors in the association between chronotype and depressive symptoms amongst young adults, using cross-sectional data collected from a university student sample (*N* =  546).

**Results:**

Evening-types had significantly higher levels of depression symptoms, poorer sleep quality, and lower levels of ‘acting with awareness’ and ‘describing’, as well as higher rumination and alcohol consumption. Mediation analyses demonstrated that the link between chronotype and depression was fully mediated by ‘acting with awareness’, ‘describing’, sleep quality, and alcohol consumption.

**Limitations:**

Only subjective measures were employed, and due to the cross-sectional design, no causal inferences can be made.

**Conclusion:**

The mediation results shed light on the crucial role of specific mindfulness facets, sleep, and alcohol consumption for explaining why evening types are at higher risk of depression; findings have import for potential interventions aiming to reduce depression risk amongst young adults in particular.

## Introduction

Morningness-eveningness (or diurnal preference) refers to an individual’s sleep-wake behaviour (preferred bed and wake times, and times preferred for peak cognitive/physical performance). Diurnal preference impacts mental health: studies have linked a tendency towards eveningness (late chronotype) to a higher risk of major depressive disorder (MDD) [1,2]. Eveningness is significantly associated with MDD even when potential confounders, e.g., sociodemographic factors, somatic health, sleep duration and insomnia ratings, are controlled for [1,3,4]. Meta-analyses have shown that evening types have higher levels of depressive symptomatology in both non-clinical and clinical populations [5,6]. In these, no difference in effect size was observed between clinical and nonclinical samples, suggesting that eveningness may raise vulnerability for depression rather than being a consequence of it. Indeed, studies have associated eveningness with neuroticism (a personality trait describing individuals who are more emotionally reactive and prone to experience negative emotions and depression), negative biases in emotional processing and impaired emotion regulation [7,8]. However, knowledge around the mechanisms linking eveningness to depression is lacking. This is of particular import given the high incidence of depression, particularly in young adult populations, with over 60% of mental health problems established by age 25 [9].

Despite being considered to be a relatively stable trait in adulthood (and with a high heritability and genetic component [10]), an individuals’ diurnal preference do tend to vary across the lifespan [11,12]; lateness tendency peaks in late adolescence/early adulthood and thus a large proportion of individuals aged 17 to 25 are typically categorised as evening-types [12,13]. Lateness tendency then tends to plateau before a gradual trend towards morningness becomes evident in later life [13]. Young adulthood, when eveningness peaks, corresponds with a higher risk of MDD symptoms compared to other age groups [14] and evening-types in this age range are at highest risk [15–17]. Adolescents with an evening preference have lower sleep quality, which can impact their cognitive, emotional and physical health [18]. Eveningness tendency also predicts depressive symptoms and MDD risk in youth when assessed longitudinally [19], supporting a possible causal relationship. In general, there is a large body of work showing an association between eveningness and both subclinical and clinical depression symptoms [20]. A meta-analysis (*N* =  15734) found consistent evidence that an eveningness orientation is associated with a higher risk of depressive symptoms [5]; only one of the 36 studies included failed to find a relationship.

To better understand the relationship between chronotype and depression, it is essential to investigate possible underlying psychological mediators. Rumination (dysfunctional, repetitive, and retrospective thoughts about negative situations or events, [21]) is one candidate. Research has shown that rumination tendency predicts future depressive symptoms in young adults [22–26]. Regarding mediators in this relationship, a study in healthy controls and individuals with a lifetime history of depression, covering the wider adult age range, found that the cognitive reactivity, and its rumination subscale in particular, was a significant mediator between chronotype and depression, with age, gender, insomnia symptoms, and neuroticism controlled for [3]. On the other hand, a study in healthy Dutch students found that evening chronotype was associated with depressive symptom severity, adjusted for age and gender, but did not find rumination to be a mediator in this relationship [27], so some clarification is needed. These studies measured rumination via a subscale of two different cognitive reactivity questionnaires (the Leiden Index of Depression Sensitivity-Revised/ Cognitive Emotion Regulation Questionnaire), which could explain the differences in findings. Here, we use a dedicated and well-validated rumination measure to clarify the role of rumination in relation to chronotype and depression.

Recently, there has been an upsurge of interest in mindfulness as a protective trait for poor mental health. The concept of mindfulness originated from Buddhist philosophy, it can be defined as maintaining a non-judgemental awareness of present experience [28]. Research has consistently shown the potential benefits of practising mindfulness on psychological health (see review by [29]) and mindfulness-based interventions have been shown to be effective in reducing depressive symptoms in student-age populations [30,31]. Mindfulness -based interventions have been proposed to reduce depressive symptoms by targeting key mechanisms such as rumination (see review [32]) and sleep quality [33–35].

At the trait level, mindfulness could be important in explaining the link between chronotype and depression risk. There is some evidence to suggest that morningness is associated with higher levels of mindfulness in healthy adults [36,37]. Marques [31] found that, in participants aged between 18-67, morning chronotypes scored significantly higher on the “acting with awareness” aspect of mindfulness, but not on other subscales from the Five Facet Mindfulness Questionnaire. However, they did not consider potential confounding variables like gender, age or sleep quality. In a predominantly female student sample, Walsh [32] found that morning chronotypes had significantly higher levels of mindfulness on the Mindful Attention Awareness Scale, but this does not provide facet-level data, and again potential cofounders were not considered.

Another potentially important mediator is alcohol consumption. Numerous studies have investigated the role of alcohol and how it affects evening-types’ mental health. Research confirmed that the evening-types show significantly higher alcohol consumption when compared to morning-types [3,38,39]. Furthermore, there is substantial body of research indicating that alcohol misuse is associated with higher risks of depression over time, and is likely bidirectional [40,41]. Therefore, understanding the inter-relationships between alcohol consumption, chronotype, and depression is important, but remains understudied. Wittman [42] showed mediating effects of substance use (alcohol and smoking) in the link between chronotype and depression; Fernando [43] did a similar analysis, and found that drug use (alcohol, smoking and cannabis) significantly mediated the link between chronotype and mental health (stress, anxiety & depression). In both of these studies, a composite measure of substance use was utilised. In contrast, the work by Van den Berg [27] in healthy Dutch students found that alcohol consumption (and not other substances) has a significant mediating effect between chronotype and depression: thus, we focused solely on alcohol use in the current work.

Finally, sleep quality plays an important role in mental health [40]. A bidirectional relationship between subjective sleep quality and depression [44–46] has been consistently shown; poorer subjective sleep quality is an antecedent of depressive symptoms in both MDD and non-clinical subjects [47]. Moreover, poorer sleep quality has been linked to rumination [48] and neuroticism [49]; but mindfulness treatments are effective in reducing sleep disturbance [50]. Evening chronotypes have consistently been shown to have overall worsened subjective sleep quality [47,51–53], serving as a likely pathway in the relationship between eveningness and depression [54]. Van den Berg [27] found the eveningness and depression link was mediated by sleep quality in their study in healthy Dutch students.

In sum, studies have shown that evening-types have higher odds of experiencing depressive symptoms, but the mediating mechanisms are currently unclear. As outlined above, evidence suggests that strong candidates include rumination, mindfulness, sleep quality and alcohol consumption, but there is no study to date that has considered all of these in a single design. The present cross-sectional study set out to explore these using a comprehensive set of self-report measures in a young adult sample. We hypothesised that: (1) Compared to morning and intermediate types, evening types will show significantly higher levels of depression, rumination, and alcohol consumption, lower levels of mindfulness, and poorer sleep quality; (2) Rumination, mindfulness facets, sleep quality and alcohol consumption, will act as mediators in the relationship between chronotype and depression symptoms. Given the high incidence of depression symptoms in young adults [55], the age point at which eveningness peaks, results could inform knowledge around underlying mechanisms and potential intervention strategies to improve young adult mental health.

## Methods

### Participants and procedure

Ethics approval was granted by the University of Surrey Ethics Committee; participants provided written informed consent through the online platform Qualtrics. The data collection period for this study started on 16^th^ April 2021 and ended on 30^th^ March 2023. The study was advertised to all undergraduate students at University of Surrey and there were no exclusion criteria. Any undergraduate could take part. It was conducted using the Qualtrics platform and took approximately 20-25 minutes in total. A total of *N* =  546 participants took part (31.5% male, 68.5% female); mean age was 19.77 years old (*SD* =  1.58), range 17 to 28. Participants received course credit for taking part.

### Measures

Subjective sleep quality was measured by the Pittsburgh Sleep Quality Index (PSQI) [56]. The PSQI is comprised of 7 components for assessing sleep quality: subjective sleep quality, sleep latency, sleep duration, sleep efficiency, sleep disturbances, use of medication, and daytime dysfunction. Global PSQI score ranges from 0 to 21, with higher scores indicating poorer sleep quality.

Chronotype was assessed with the reduced version of the Morningness-Eveningness Questionnaire (rMEQ) [57], a self-report questionnaire consisting of 5 items derived from the original 19 questions. The rMEQ comprises questions about the sleep-wake cycle, subjective energy peak time, as well as self-evaluation of chronotypes. The cut-offs for chronotype categories are: scores 4-11 indicating eveningness; 12-17 indicating an intermediate type and 18-25 indicating morningness, these cut-offs have been validated against circadian motor activity [58]. The validity and reliability of the rMEQ has been well demonstrated [58,59].

Rumination was measured using the rumination subscale of the Rumination-Reflection Questionnaire (RRQ) [60]. Participants indicated their agreement with statements such as ‘I spend a great deal of time thinking back over my embarrassing or disappointing moments’ (rumination subscale). The RRQ has excellent internal consistency [60].

Depression was measured using the Hospital Anxiety and Depression Scale (HADS) [61]. This questionnaire consists of 14 items and two subscales, measuring depression and anxiety levels.

Five Facets of Mindfulness Questionnaire Short Form (FFMQ-SF) was used to measure mindfulness levels [62]. The questionnaire contains 24 items with 4 to 5 questions for each facet of mindfulness: Observing, Describing, Acting with awareness, non-Judging to inner experience and non-Reactivity to inner experience.

Alcohol consumption was assessed by asking participants how many units of alcohol they drank per week. A unit was defined as a small glass of wine or half a pint of lager. Participants selected one of the following answers: none, 1 to 5, 6 to 10, 11 to 15, 16 to 20, and more than 20. Then participants categorised according to alcohol consumption (units per week) with scores ranging from 1 to 6. Higher score indicates higher alcohol consumption.

### Statistical approach

All data were analysed using the Statistical Package for Social Science (SPSS version 27). To test the first hypothesis, a series of non-parametric ANCOVA were conducted with age and sex included as covariates. A Quade non-parametric ANCOVA was chosen due to the failure in meeting the assumptions of parametric ANCOVA and unequal sample sizes between chronotype groups. Bonferroni corrections were applied in the planned comparisons which tested the hypothesis that evening types will differ in their scores compared to morning/intermediate types [63]. For Hypothesis 2, a parallel mediation analysis with multiple mediator variables was conducted using PROCESS model 4, with bootstrapping (10000 samples). Bias-corrected bootstrap confidence intervals are reported for all indirect effects and used to assess significance [64]; standardized coefficients are reported for all.

## Results

### Data pre-processing: Assumption testing

The appropriateness of covariates was checked. Firstly, covariates should be related to the dependent variables [65]. A Pearson correlation analysis was conducted, and it was shown that age and sex did correlate with multiple dependent variables, and thus, they were included as covariates.

### Participant characteristics

The majority of participants were classified intermediate (*N* =  256) and evening chronotype (*N* =  252), and only 38 were classified morning chronotype. Participant characteristics were analysed using Kruskal Wallis for age, and chi-square for sex distribution across chronotype groups. Results showed that there were no significant age (*p* = .051) and sex (*p* = .098) differences across the chronotype groups.

### Effects of chronotype

Table 1 presents the descriptive statistics, by chronotype group (Evening/Intermediate/Morning). A series of Quade non-parametric ANCOVA were conducted to examine the effect of chronotype category on depressive symptoms, rumination, sleep quality, mindfulness facets, and alcohol consumption (Hypothesis 1). To explore group differences, planned comparisons were conducted, contrasting evening vs intermediate types, and evening vs. morning types, with a Bonferroni-corrected p value threshold of (0.05/8) =  0.00625 (see Table 1).

**Table 1:** Descriptive statistics and non-parametric ANCOVA results.

	Evening chronotypes	Intermediate chronotypes	Morning chronotypes			
	*N* = 252	*N* = 256	*N* = 38			
	*MDN* (*IQR*)	*MDN* (*IQR*)	*MDN* (*IQR*)	*F*	*p*	Significant Contrasts
Depressive Symptoms	7.00 (5.00)	5.00 (6.00)	5.00 (5.50)	5.23	**.006**	Evening**> **Intermediate
Rumination	47.00 (11.75)	47.00 (11.00)	45.50 (11.50)	1.30	.27	–
Sleep Quality	8.00 (5.00)	7.00 (5.00)	5.00 (4.00)	10.34	**<.001**	Evening**> ** Morning Evening**> ** Intermediate
Alcohol consumption	2.00 (2.00)	2.00 (2.00)	1.00 (1.00)	13.44	**<.001**	Evening**> ** Morning Evening**> ** Intermediate
FFMQ						
Describing	16.00 (6.00)	16.00 (5.00)	18.00 (6.25)	2.40	.09	–
Observing	14.00 (4.00)	14.50 (4.00)	13.50 (5.00)	2.35	.10	–
Acting with awareness	15.00 (5.00)	15.00 (5.00)	17.00 (5.25)	5.82	**.003**	Evening **< **Morning
Non-Judging to inner experience	13.00 (5.00)	14.00 (5.00)	14.00 (5.25)	1.20	.30	–
Non-Reactivity to inner experience	13.00 (5.00)	14.00 (5.00)	15.00 (5.25)	1.49	.23	–

FFMQ; Five Facet Mindfulness Questionnaire.

A Quade non-parametric ANCOVA showed that there was a significant difference in depressive symptoms between chronotype categories, controlling for age and sex; planned comparisons showed that evening types had significantly higher depressive symptoms than intermediate types (*p* = .002). No significant differences emerged between evening and morning types.

Regarding the FFMQ facets, the effect of chronotype was significant only for ‘acting with awareness; planned comparisons showed that morning types had significantly higher scores on the acting with awareness subscale (*p* = .002) than evening-types. No significant differences found between evening and intermediate types. There was no significant effect of chronotype on rumination.

A significant effect of chronotype was also emerged for sleep quality, showing that evening chronotypes had worse sleep quality than intermediate (*p* < .001) and morning (*p* < .001) chronotypes.

Finally, there was a significant effect of chronotype on alcohol consumption; planned comparisons indicated that evening chronotypes consumed higher units of alcohol compared to intermediate (*p* = .001) and morning (*p* < .001) chronotypes.

### Mediation analyses

First, correlations were assessed between all variables of interest (see Table 2). For these, rather than using categorical chronotype (as above), the actual continuous rMEQ score was used. Total rMEQ score was significantly correlated with FFMQ total score and most of its subscales, as well as with depression symptoms, alcohol consumption, sleep quality, and rumination.

**Table 2:** Pearson correlations among study variables.

Variables	1	2	3	4	5	6	7	8	9	10
1. Chronotype (score)	-									
2. Describing	.15***	-								
3. Observing	.11**	.11^*^	-							
4. Acting with Awareness	.21***	.43***	−.06	-						
5. Non-judging to inner experience	.11*	.28***	−.12**	.44***	-					
6. Non-reactivity to inner experience	.12**	.29***	.07	.20***	.31***	-				
7. Rumination	−.13**	−32***	.08	−.42***	−.56***	−.53***	-			
8. Sleep quality	−.27***	−.38***	.02	−.37***	−.34***	−.33***	.36***	-		
9. Depression	−.18***	−.36***	−.01	−.40***	−.32***	−.25***	.29***	.44***	-	
10. Alcohol Consumption	−.19***	.03	−.05	−.02	−.03	−.02	.03	−.002	−.10*	-

***p < .001,

**p < .01,

*p < .05.

A single parallel mediation model (*N* =  546) was performed using PROCESS; the outcome variable was depression symptoms (from HADS), and the predictor variable was rMEQ total score. All FFMQ subscales, rumination, sleep quality, and alcohol consumption were included simultaneously as mediators; the model controlled for age and sex, see Table 3. Total effect of chronotype on depressive symptoms was significant (Effect =  -.17, p < .001), while the direct effect of chronotype on depressive symptoms was non-significant (Effect =  - 0.04, *p* = .31) indicating that a full mediation occurred. Significant indirect effects were seen for the FFMQ subscales “acting with awareness” (Effect =  -0.04, 95% CI: -0.06 to -0.02), and “describing” (Effect =  - 0.02, 95% CI: - 0.04 to - 0.01). Sleep quality (Effect =  -0.07, 95% CI: -0.10 to -0.04), and alcohol consumption (Effect =  0.02, 95% CI: 0.01 to 0.04) were also significant mediators. The mediating effects of observing, non-judgement, non-reactivity, and rumination were non-significant (Table 3).

**Table 3:** Summary of mediation analysis between the relationship of chronotype (IV) and depression (DV).

	Effect of IV on M (a)			Effect of M on DV (b)			Direct effect (c’) of IV on DV			Indirect effect of IV on DV (ab)	
Mediator	*Effect (SE)*	*t*	*p*	*Effect (SE)*	*t*	*p*	*Effect (SE)*	*t*	*p*	*Effect (SE)*	*95% CI (ab)*
Describing	0.15 (0.04)	3.45	**.001**	−0.15 (0.04)	−3.78	**<.001**	−0.04 (0.04)	−1.02	.31	−0.02 (0.01)	**−0.04 to −0.01**
Observing	0.11 (0.04)	2.65	**.008**	−0.02 (0.04)	−0.68	.50	−0.04 (0.04)	−1.02	.31	−0.003 (0.004)	−0.01 to 0.01
Acting with Awareness	0.21 (0.04)	4.91	**<.001**	−0.19 (0.04)	−4.42	**<.001**	−0.04 (0.04)	−1.02	.31	−0.04 (0.01)	**−0.06 to −0.02**
Non-judgement	0.10 (0.05)	2.38	**.018**	−0.09 (0.04)	−1.6	.051	−0.04 (0.04)	−1.02	.31	−0.01 (0.01)	−0.02 to 0.0001
Non-Reactivity	0.13 (0.04)	3.01	**.003**	−0.06 (0.04)	−1.44	.15	−0.04 (0.04)	−1.02	.31	−0.01 (0.01)	−0.02 to 0.002
Rumination	−0.13 (0.10)	−2.93	**.004**	0.02 (0.02)	.55	.58	−0.04 (0.04)	−1.02	.31	−0.003 (0.01)	−0.02 to 0.01
Sleep Quality	−0.27 (0.04)	−6.48	**<.001**	0.25 (0.04)	6.19	**<.001**	−0.04 (0.04)	−1.02	.31	−0.07 (0.02)	**−0.10 to −0.04**
Alcohol consumption	−0.18 (0.02)	−4.27	**<.001**	−0.11 (0.10)	−3.23	**.001**	−0.04 (0.04)	−1.02	.31	0.02 (0.01)	**0.01 to 0.04**

All effects reported above are the completely standardised coefficients their Standard Errors (SE), For the indirect effects, 95% bootstrapped Confidence Intervals (CI) are reported. Bold type indicates significance.

## Discussion

We examined the effect of chronotype in relation to depression symptomatology in young adults and assessed the potential mediating effect of key factors identified by previous research. The role of chronotype, mindfulness, and rumination in relation to depression risk is well established [1,3,36], but studies that comprehensively explore chronotype effects on depression risk and mediating factors are lacking, particularly amongst young adults. Thus, the current study set out to investigate the psychological mechanisms that could explain why evening-types are at higher risk of depression. This is of importance given the high (and increasing) prevalence of depression and anxiety amongst young adults: a better understanding of mechanisms could serve to inform intervention strategies [55].

As expected, eveningness tendency was common in the sample, with nearly half of the participants classified as evening types, in line with previous work in this age range [13,66]. Evening chronotypes had significantly more depressive symptoms when compared to morning and intermediate chronotypes, again consistent with previous studies [67–69]. Also, in line with previous findings [3], evening chronotypes reported significantly higher levels of rumination when compared to morning chronotypes. A likely explanation is a tendency for rumination to occur at the end of the day rather than in the morning [70]. Since evening chronotypes have a delayed circadian rhythm and stay awake later into the night, this gives them extra time to ruminate, potentially increasing their risk of depressive symptomatology [3].

There has been significant recent research interest into mindfulness as a potential protective trait or skill, for addressing mental health issues in youth [71]. Various studies have reported correlations between self-reported mindfulness and indices of psychological health [29]. These include depression [72,73], neuroticism [74,75], rumination and difficulties with emotion regulation [76]. Here, chronotype correlated with levels of trait mindfulness, as has also been shown by the limited previous work on this [36,37]. To date, only one previous study has examined relationships between chronotype and the individual facets: Marques [36] linked morningness to higher “acting with awareness” in a sample covering the whole adult age range. In the current study, we also found an effect of chronotype on “acting with awareness”, with morning types scoring higher on this facet compared to evening types. ‘Acting with awareness’ is the ability to be non-judgemental, and pay attention to both positive or negative emotions and thoughts with equanimity [76]. This is in accordance with previous studies exploring links between mindfulness facets and mental health: a longitudinal study in adolescents found that ‘acting with awareness’ in particular predicted a reduction in depression at 4-month follow-up [77]. Likewise, Calvete [78] found that the ‘acting with awareness’ facet moderates predictive associations between stressors and various psychological symptoms at one-year follow-up, also amongst adolescents. A cross-sectional study in young adults found ‘acting with awareness’ to be the only facet that showed significant direct effects on various health outcomes, which included depression, anxiety, perceived stress and alcohol-related problems [79].

Results also indicated a significant difference in sleep quality between chronotypes, evening chronotypes had significantly poorer sleep quality, as has been shown consistently by previous studies [51,80,81]. This can be explained by social jet lag: since evening chronotypes’ biological rhythm is misaligned with daily work/study routines, their sleep quality necessarily suffers and a ‘sleep debt’ is accumulated [82]. Sleep debt, as well as daytime sleepiness, has been shown to mediate the relationship between eveningness and depression among young adult university students [83].

We found alcohol consumption to be significantly higher in evening-types, as documented in previous work such as that by Wittmann et al. [42], who suggest that this is because alcoholic beverages are often consumed late at night rather than early in the day.

We then conducted a parallel mediation model with chronotype as a continuous predictor variable and depressive symptoms as the outcome variable, to test the mediating role of mindfulness facets, rumination, sleep quality, and alcohol consumption in the relationship between chronotype and depression. This has not been explored previously, and we found a full mediation effect, driven by sleep quality, ‘acting with awareness’, ‘describing’, and alcohol consumption. Sleep quality explained the most variance as a mediator, followed by ‘acting with awareness’.

Although prior research has shown morning types to have higher levels of mindfulness [36,37], this is the first study to explore the potential mediating effect of mindfulness and its facets on the link between chronotype and depression symptoms. Gorgol [84] found that trait mindfulness moderated the link between eveningness and both depressive and anxiety symptoms; the strength of the relationship decreased with higher dispositional mindfulness. The current study provides more detailed insights by considering the facets of mindfulness, which points to ‘acting with awareness’ as being of particular import [84]. Marques [36] suggest that morning types present with higher ‘acting with awareness’ because of their tendency to sleep better and therefore have a lower risk of fatigue, inattention and mind wandering during the day. On the other hand, evening chronotypes usually have poorer sleep quality, causing them to be more prone to daytime sleepiness and thus less awareness of the present moment. Several studies have shown poorer attention abilities in evening-types, supporting this assertion [85,86]. Further work (e.g., with neurocognitive testing) is needed to directly explore this possibility. In terms of the link to mental health, one explanation could involve how individuals relate to the present, past and future. Depressive and anxiety symptoms are associated with past-negative and present-fatalistic time perspectives, and studies have linked eveningness with past-negative and present-fatalistic time perspectives, while morningness has been linked to a more a positive and future-oriented time perspective [87,88], possibly as a result of better emotional regulation. “Acting with awareness” encourages a focus on the present moment, thus reducing the amount of attention towards negative aspects of the past, present fatalism, and negative anticipation of the future [89]; this can act to reduce the risks of experiencing depressive symptoms by promoting a more balanced time perspective [90], and this also merits further study.

We also found that the ‘describing’ facet also mediated the relationship between chronotype and depression, albeit to a lesser extent than ‘acting with awareness’. ‘Describing’ emotions and situations could be beneficial to regulate the intensity and valance of emotions, as labelling emotions is associated with reduced neural and physiological reactivity to emotional stimuli [91,92]. Thus, those higher on the describing facet might have less prolonged emotional reactivity, which has been directly associated to higher levels of depressive symptoms [93].

Our results demonstrated that subjective sleep quality also mediated the relationship between eveningness and depression, as shown in previous literature [27,94,95]. The incompatibility between evening chronotype’s internal clock and work/school schedule (social jet-lag) is associated with sleep disturbances and often leads to lower sleep quality, increased sleep debt and daytime sleepiness [82]; this has previously been shown to mediate the relationship between chronotype and depression [83]. Also, as a result of a delayed sleep-wake schedule, evening-types are likely to encounter reduced light exposure, which can have an impact on mood [96]. A similar mechanism has been proposed to explain seasonal depression [97].

Lastly, our results showed alcohol consumption also mediates the link between chronotype and depressive symptomatology. Surprisingly, alcohol consumption was found to have a protective mediating effect. Previous research generally suggests that alcohol use links to lower mood [27,42,98]; the opposite was found here. However, in our sample, while the evening chronotypes drank more than the intermediate and early types, only 8.3% of evening types reported more than 15 units weekly consumption. This suggests that even the evening-types in our sample only drink in moderation and are unlikely to engage in binge drinking. Townshend [99] found evidence that only problematic alcohol use is associated with lower mood. Van den Berg [27] found similar results to us in their study of undergraduate students: evening chronotypes drank more, but more alcohol consumption was associated with less depressive symptoms cross-sectionally. Social connectedness and social support are key protective factors against depression in youth [100]. College students often use alcohol as a social lubricant, enhancing their social life [101]. Future work should thus measure social factors in order to confirm this protective association.

### Limitations

Firstly, the cross-sectional design of the present study precludes conclusions regarding causality. In addition, when constructing the mediation models, we assumed a causal process between chronotype and depression (first, chronotype is affecting the mediators, and then the mediators affect depression risk). This was justified by chronotype being a generally stable ‘trait-like’ construct, while depression symptoms fluctuate and can be regarded as an outcome variable. However, it is likely these two constructs have some element of reciprocal bidirectionality, and mediators, e.g., mindfulness might, in turn, be exerting some effects on chronotype. Secondly, only subjective measures were employed in this study. While well-validated measures were selected, recall and social desirability bias could have affected the data. In particular, objective measures of sleep and chronotype would be useful in future work to confirm the current findings. Thirdly, the majority of our sample were female. Sex differences in chronotype are well established; in youth, women are more likely to be morning-oriented than men [102], which has biological underpinnings [103]. Further, depression and chronotype are both significantly linked to gender: there is some evidence that risk of depression has a stronger link with evening chronotype in women [104]. Moreover, depression is more common in women than in men, while (as found here) evening chronotype is more common in men than in women [104]. We controlled for sex in our analyses, but future research should recruit more males and test sex effects explicitly. Finally, the sample was derived from an undergraduate University population via self-selection sampling, and therefore might not be representative of the general population, future work should address this to confirm generalisability of findings: studies should also investigate whether the relationships observed here are generalisable across the lifespan since the current study focused only on young adults, when eveningness tendency is more common than in later life.

### Conclusion

The present study comprehensively explored the relationship between chronotype and depressive symptoms, with a focus on mindfulness, rumination, alcohol consumption and sleep quality as mediators. In line with previous literature, chronotype had a significant effect on depression, mindfulness, rumination, alcohol consumption and sleep quality. However, this is the first study to test these as mediators, and a full mediation was found, with sleep quality, mindfulness, and alcohol consumption found to be significant mediators. This adds valuable mechanistic detail to the literature, showing that these factors explain the relationship between evening chronotype and depression risk in young adults. Importantly, we considered the different facets of mindfulness as factors. Results pointed specifically to a protective role of the ‘acting with awareness’ facet. Results inform potential intervention strategies for addressing the highly problematic levels of mental health issues currently affecting young adults worldwide. Given the growing popularity of mindfulness training for improving mental health, the current results indicate that enhancing the ‘acting with awareness’ facet of mindfulness should be prioritised in the design of mindfulness interventions, to maximise efficacy.

## Supporting information

S1 File Dataset.
(XLSX)

S2 File Metadata.
(DOCX)
