# Supplementary material for: Mindfulness mediates the association between chronotype and depressive symptoms in young adults
Source: PLoS One. 2025 Mar 19;20(3):e0319915. doi: 10.1371/journal.pone.0319915 (PMC11922220; doi:10.1371/journal.pone.0319915)
Supplement: S2 File — (DOCX) [file pone.0319915.s002.docx]

Metadata

**Age:** Participant’s age.

**Gender:** Male gender was coded as 1, and female gender was coded as 2.

**Alc_Cons** represents the level of weekly alcohol consumption which was coded from 1 to 6 based on participant’s weekly alcohol consumption (as explained in the method section).

**HADS_A** represents the anxiety scores based on the Hospital Anxiety and Depression Scale.

**HADS_D** represents the depressive symptoms scores based on the Hospital Anxiety Depression Scale.

**The columns of DES_SUB (describing), OBS_SUB (observing), AWA_SUB (acting with awareness), NJ_SUB (non-judgment), and NR_SUB (non-reactivity)** represent five facets of mindfulness, which was calculated based on Five Facet Mindfulness Questionnaire (24-item Short Form).

**T_FFMQ** represents the total scores of all five mentioned facets above to gain total mindfulness score.

**rMEQ_T** represents the total scores of reduced Morningness Eveningness Questionnaire (rMEQ).

**EMgroup** represents chronotypes groups (evening/intermediate/morning) based on the cut off scores on rMEQ.

**PSQI_T** represents total sleep quality scores based on Pittsburgh Sleep Quality Index (PSQI),

**Tot_RUM** represents the total scores of rumination subscale of Rumination-Reflection Questionnaire.
