# Supplementary material for: Mindfulness mediates the association between chronotype and depressive symptoms in young adults
Source: PLoS One. 2025 Mar 19;20(3):e0319915. doi: 10.1371/journal.pone.0319915 (PMC11922220; doi:10.1371/journal.pone.0319915)
Supplement: Attachment — Submitted filename: Response to Reviewers.docx [file pone.0319915.s003.docx]

Additional Editor Comments:

The study methods description should be better elaborated. In specific, information about study population, incl. source, target population; eligibility, inclusion and exclusion criteria. How the eligible subjects were invited, how many were eligible and how many participated (% of the eligible). Was the selection bias excluded? The limitation described in the discussion should consider also potential for selection bias and whether it was excluded.

**Thank you for this- we have added detail in the Methods, and in the Limitations.**

Reviewers' comments:

Reviewer's Responses to Questions

**Comments to the Author**

1. Is the manuscript technically sound, and do the data support the conclusions?

The manuscript must describe a technically sound piece of scientific research with data that supports the conclusions. Experiments must have been conducted rigorously, with appropriate controls, replication, and sample sizes. The conclusions must be drawn appropriately based on the data presented.

Reviewer #1: Partly

Reviewer #2: Partly

2. Has the statistical analysis been performed appropriately and rigorously?

Reviewer #1: Yes

Reviewer #2: Yes

3. Have the authors made all data underlying the findings in their manuscript fully available?

The [PLOS Data policy](https://eur02.safelinks.protection.outlook.com/?url=http%3A%2F%2Fwww.plosone.org%2Fstatic%2Fpolicies.action%23sharing&data=05%7C02%7Cgy00111%40surrey.ac.uk%7C2f8f7da4a32149c0e69808dca646c6f6%7C6b902693107440aa9e21d89446a2ebb5%7C0%7C0%7C638568070117941140%7CUnknown%7CTWFpbGZsb3d8eyJWIjoiMC4wLjAwMDAiLCJQIjoiV2luMzIiLCJBTiI6Ik1haWwiLCJXVCI6Mn0%3D%7C0%7C%7C%7C&sdata=Cyroxdrr8uXqmNZI8cQiyqxIF0l265DcdTqQpIkhLso%3D&reserved=0) requires authors to make all data underlying the findings described in their manuscript fully available without restriction, with rare exception (please refer to the Data Availability Statement in the manuscript PDF file). The data should be provided as part of the manuscript or its supporting information, or deposited to a public repository. For example, in addition to summary statistics, the data points behind means, medians and variance measures should be available. If there are restrictions on publicly sharing data—e.g. participant privacy or use of data from a third party—those must be specified.

Reviewer #1: Yes

Reviewer #2: No

4. Is the manuscript presented in an intelligible fashion and written in standard English?

PLOS ONE does not copyedit accepted manuscripts, so the language in submitted articles must be clear, correct, and unambiguous. Any typographical or grammatical errors should be corrected at revision, so please note any specific errors here.

Reviewer #1: Yes

Reviewer #2: No

5. Review Comments to the Author

Please use the space provided to explain your answers to the questions above. You may also include additional comments for the author, including concerns about dual publication, research ethics, or publication ethics. (Please upload your review as an attachment if it exceeds 20,000 characters)

Reviewer #1: My main concern about the article is that a large number of potential mediators was considered. Thus, the aim and hypothesis is quite scattered into different areas, i.e. psychological traits (mindfulness, tendency to rumination), sleep quality and lifestyle factors (alcohol consumption), but those variables are treated equally. Also, as state, a parallel mediation analysis was conducted - as I understand, separate models were constructed for each potential mediator. Please qualify.

I suggest to reduce the number of potential variables of interest (mediators) and run a simultaneous, not parallel mediation analysis, to include all the potential mediatiors in one model and thus to better elucidate which factors exactly are responsible for the total mediation between chronotype and depressive symptoms. I believe the sample size is enough to run such an analysis, especially that the bootstrapping is stated to be utilized. This would improve the quality of this research. The discussion should be updated accordingly then.

**Thank you for raising this and apologies for any lack of clarity. The mediating roles of variables were assessed simultaneously using a single parallel mediation model (Hayes Model 4) by including all variables as mediators in the model. We have now made this clearer throughout that the multiple mediators were assessed simultaneously.**

Minor issues:

Table 2 is a bit hard to read - I suggest removing the exact p values and marking the significant correlations with symbols for thresholds p<0.05, p<0.01 and p<0.001.

**Thank you. We have now removed p-values from table and used symbols to indicate significance.**

The utilized abbreviations are not explained in the Tables.

**Thank you, we have now added the explanation for abbreviations used in the tables as a note below the table where necessary.**

I am not sure if marking the indirect effect of IV on DV with „a x b” is correct, because this denotations suggests a moderation (or interaction), not mediation. Please verify this.

**Thank you for the comment. We have now changed to this to “ab” to represent the indirect effect.**

What is the measure of the Effect in the mediation analysis? Is it a standardised or non-standardises parameter? Please clarify, I suggest presenting the standardised parameters to enable interpratation in terms of effect size.

**Thank you for raising this. We have now made clear in the method section that all are standardized coefficients.**

I also have doubt whether the table contains the parameters resulted from bootstrapping - usually Bootstrap provides effects with their effect sizes, but not p values. Consequently, the statistical significance of the associations is assessed by confidence intervals, not p values. Please verify this and explain.

**Thank you for this comment, we report the bootstrap confidence intervals for all indirect effects, and this is now specified in the method and results sections.**

Reviewer #2: 1. While analyzing association between chronotype and depressive symptoms is a worthwhile topic, my biggest concern refers to mechanism that the Authors claim to have been proven by previous and the current research. Overall, I disagree with the causality imposed by the Authors in multiple places, from title to discussion. The are no proofs that evening chronotype leads to depression and the opposite direction is similarly likely. Therefore, I urge the Authors to be more caution in these statements and modify them so that researchers’ intepretations are separated from studies results. The cross-sectional desing of this study excludes possibility to analyze true mediation [as opposed to statistical – in fact confounder analysis in this study], and is even more undermined by the Authors’ plan to “explore” [mediation in cross-sectional data can be inferred only if there is a strong background proving causality in links between the studied variables, e.g., chronotype->mindfulness). So, I urge tha Authors to remove all statements imposing causality, stating this is a confounder analysis, while causality mechanism can be suggested as a part of introduction/discussion, but not as a proved fact, bur rather a speculation.

**We thank the reviewer for raising this important point. For brevity, the introduction did not fully set out the evidence supporting a causal relationship between evening chronotype and depression and we apologise for the omission, this is of course essential given that the mediation analysis is predicated upon it. There is considerable evidence showing higher risk amongst evening types, for example:**

**Kivelä, L., Papadopoulos, M. R. & Antypa, N. Chronotype and psychiatric disorders. Curr. Sleep Med. Rep.** [**https://doi.org/10.1007/s40675-018-0113-8**](https://doi.org/10.1007/s40675-018-0113-8) **(2018).**

**Taylor, B. J. & Hasler, B. P. Chronotype and mental health: Recent advances. Curr. Psychiatry Rep. 20, 59 (2018).**

**Kitamura, S. et al. Evening preference is related to the incidence of depressive states independent of sleep-wake conditions. Chronobiol. Int. 27, 1797–1812 (2010).**

**Merikanto, I. et al. Evening types are prone to depression. Chronobiol. Int. 30, 719–725 (2013).**

**Hidalgo, M. P. et al. Relationship between depressive mood and chronotype in healthy subjects. Psychiatry Clin. Neurosci. 63, 283–290 (2009).**

**Antypa, N., Vogelzangs, N., Meesters, Y., Schoevers, R. & Penninx, B. W. J. H. Chronotype associations with depression and anxiety disorders in a large cohort study. Depress. Anxiety https://doi.org/10.1002/da.22422 (2015).**

**Merikanto, I. et al. Circadian preference links to depression in general adult population. J. Affect. Disord. 188, 143–148 (2015).**

**Chan, J. W. Y. et al. Eveningness and insomnia: Independent Risk factors of nonremission in major depressive disorder. Sleep https://doi.org/10.5665/sleep.3658 (2014).**

**Gaspar-Barba, E. et al. Depressive symptomatology is influenced by chronotypes. J. Affect. Disord. 119, 100–106 (2009).**

**Bauducco, S., Richardson, C. & Gradisar, M. Chronotype, circadian rhythms and mood. Curr. Opin. Psychol. 34, 77–83 (2020).**

**Taillard, J., Sagaspe, P., Philip, P. & Bioulac, S. Sleep timing, chronotype and social jetlag: Impact on cognitive abilities and psychiatric disorders. Biochem. Pharmacol. 1, 114438. https://doi.org/10.1016/j.bcp.2021.114438 (2021).**

**While there is no doubt that depression is associated with sleep disturbance (and likely bidirectional for that relationship), we are not aware of any studies suggesting that depression can itself affect chronotype. Further, it should be mentioned that diurnal preference is considered a relatively stable trait in adulthood. Twin studies indicate heritability estimates between 46 and 57% for diurnal preference (Barclay, N. L., Watson, N. F., Buchwald, D. & Goldberg, J. Moderation of genetic and environmental influences on diurnal preference by age in adult twins. Chronobiol. Int. 31, 222–231 (2014); Barclay, N. L., Rowe, R., O’Leary, R., Bream, D. & Gregory, A. M. Longitudinal stability of genetic and environmental influences on the association between diurnal preference and sleep quality in young adult twins and siblings. J. Biol. Rhythms 31, 375–386 (2016)). Given its trait-like characteristics, this further supports treating chronotype as the causal factor in the relationship, rather than depression.**

**We have expanded the Introduction to justify our approach and we hope that this now makes the rationale clearer.**

**Line 72:**

**In general, there is a large body of work showing an association between eveningness and both subclinical and clinical depression symptoms (16). A meta-analysis (n =15734) found consistent evidence that an eveningness orientation is associated with a higher risk of depressive symptoms (5); only one of the 36 studies included failed to find a relationship.**

2. Division into chronotype groups is incorrect – the Authors analyze continuous variable and it should be analyzed in its full spectrum instead of creating artifical groups based on arbitrary derived cutoff values that result great size inbalance of the groups.

**For the first hypothesis, we compare between chronotype groups, but this is based on the well-established (and well-used) cut-offs for the MEQ (not arbitrary/derived values): these cut-offs have been externally validated against circadian motor activity. We now add more detail in the Measures section. For the mediation analysis, this is based on the continuous variable; again we now make this clearer throughout.**

3. The Authors only briefly mention sex differences in chronotype. They need to discuss them in more detail, indicating that they are well-documented [https://doi.org/10.1080/07420528.2019.1585867] and have biological underpinnings [https://doi.org/10.1016/j.psyneuen.2019.05.027].

**Thank you. We have now expanded on sex differences in chronotype, and added these references, in discussion (line 389)**

4. The term “circadian preference” semantically is incorrect, please use morningness-eveningness or chronotype or time-of-day preference.

**Thank you. We have now changed “circadian preference” to “morningness-eveningness” or “chronotype” throughout the manuscript.**

5. It is odd that the Authors analyzed planned contrasts for exploaratory purposes without hypotheses. They should rather apply bonferroni correction for multiple comparisons.

**Thank you for this.** **In hypothesis 1, we specifically hypothesised “Compared to morning and intermediate types, evening types will show significantly higher levels of depression, rumination, and alcohol consumption, lower levels of mindfulness, and poorer sleep quality”, thus, the analysis approach we use (planned contrasts with Bonferroni correction) is appropriate, please see Methods.**

6. When writing about negative bias in chronotypes no reference is enclosed. Please provide a reference [https://doi.org/10.1007/s11920-018-0925-8]

**Thank you for raising this. We have now added this reference as well as one other study which shows negative bias in late chronotype compared to intermediate and morning chronotypes (line 61).**

7. Please be consistent in using sex/gender [only one of this – sex I believe is more valid here]

**Thank you. We have now changed “gender” to “sex” where we discuss our sample/results.**

8. Proofreading by a native English speaker is advised [e.g., “an morningness”]

**Thank you for this. We have checked through and corrected.**
