# Supplementary material for: Mindfulness mediates the association between chronotype and depressive symptoms in young adults
Source: PLoS One. 2025 Mar 19;20(3):e0319915. doi: 10.1371/journal.pone.0319915 (PMC11922220; doi:10.1371/journal.pone.0319915)
Supplement: Attachment — Submitted filename: Response_to_reviewers_auresp_2.docx [file pone.0319915.s004.docx]

Reviewers' comments:

Reviewer's Responses to Questions

**Comments to the Author**

1. If the authors have adequately addressed your comments raised in a previous round of review and you feel that this manuscript is now acceptable for publication, you may indicate that here to bypass the “Comments to the Author” section, enter your conflict of interest statement in the “Confidential to Editor” section, and submit your "Accept" recommendation.

Reviewer #2: All comments have been addressed

Reviewer #3: All comments have been addressed

2. Is the manuscript technically sound, and do the data support the conclusions?

The manuscript must describe a technically sound piece of scientific research with data that supports the conclusions. Experiments must have been conducted rigorously, with appropriate controls, replication, and sample sizes. The conclusions must be drawn appropriately based on the data presented.

Reviewer #2: Yes

Reviewer #3: Yes

3. Has the statistical analysis been performed appropriately and rigorously?

Reviewer #2: Yes

Reviewer #3: Yes

4. Have the authors made all data underlying the findings in their manuscript fully available?

The [PLOS Data policy](https://eur02.safelinks.protection.outlook.com/?url=http%3A%2F%2Ftrack.editorialmanager.com%2FCL0%2Fhttp%3A%252F%252Fwww.plosone.org%252Fstatic%252Fpolicies.action%2523sharing%2F1%2F010f0193d2a6424a-7e8428ec-29d4-4aaf-8ca9-6f6f1475111e-000000%2FM1Of4NGUSZMt4-788HmgN78QVU3D5WJCNyR0MQCiAyk%3D189&data=05%7C02%7Cgy00111%40surrey.ac.uk%7C5fc46250602f4a30b87d08dd1e4a7725%7C6b902693107440aa9e21d89446a2ebb5%7C0%7C0%7C638700027378861081%7CUnknown%7CTWFpbGZsb3d8eyJFbXB0eU1hcGkiOnRydWUsIlYiOiIwLjAuMDAwMCIsIlAiOiJXaW4zMiIsIkFOIjoiTWFpbCIsIldUIjoyfQ%3D%3D%7C0%7C%7C%7C&sdata=4bXuhWMYyEe64PFqrz3KbkaDiRSL10u6bU1wdwIcJc4%3D&reserved=0) requires authors to make all data underlying the findings described in their manuscript fully available without restriction, with rare exception (please refer to the Data Availability Statement in the manuscript PDF file). The data should be provided as part of the manuscript or its supporting information, or deposited to a public repository. For example, in addition to summary statistics, the data points behind means, medians and variance measures should be available. If there are restrictions on publicly sharing data—e.g. participant privacy or use of data from a third party—those must be specified.

Reviewer #2: Yes

Reviewer #3: Yes

5. Is the manuscript presented in an intelligible fashion and written in standard English?

PLOS ONE does not copyedit accepted manuscripts, so the language in submitted articles must be clear, correct, and unambiguous. Any typographical or grammatical errors should be corrected at revision, so please note any specific errors here.

Reviewer #2: Yes

Reviewer #3: Yes

6. Review Comments to the Author

Please use the space provided to explain your answers to the questions above. You may also include additional comments for the author, including concerns about dual publication, research ethics, or publication ethics. (Please upload your review as an attachment if it exceeds 20,000 characters)

Reviewer #2: Thanks for your work. All comments I raised in my review have been adressed by the Authors adequately.

Reviewer #3: This scholarly article is well-written, written in plain language, well-structured and its analyses are sound, however I do have critical feedback to help inform potential publication at the current stage in the review process.

**We thank the reviewer for their positive comments and helpful feedback.**

“a large proportion of individuals aged 17 to 25 are typically categorised as evening-types” The authors ought to directly evaluate the validity of their findings based on this item of demographic information in the form of a statement of limitation, is the effect being examined a potential function of observer bias, p-fishing or a direct contribution from another extraneous, likely neurophysiological factor such as hormone uptake, eye movements and photon displacement, etc., more suitable for experimental analysis and scientific contribution based on the phenomenon in question? Eveningness preference especially is heavily empirically and ex post facto a less mindful choice than any other form of chronicity, thus potentially disrupting the reliability of the reporting, even if it is explored later in the background evidence in terms of mindfulness chronicity is not a priori a direct function of mindfulness but of biology.

**Indeed, chronicity is largely biologically determined. Apologies if this is not clear, but we are not at all suggesting that mindfulness impacts chronicity – we present mindfulness as an independent factor that could explain the relationship between evening chronicity and poorer mental health. We have added extra detail at the start of the 2^nd^ paragraph emphasise the biological contribution to chronicity.**

**Reference 10 has been added.**

As another reviewer commented in a previous review there is indeed a potentially exaggerated extent of causal inference being made in this paper in particular about the mediating properties of the phenomenon of diurnal preference reported to be endemic, not abnormal, in its sampled population. At the very least I would expect some background comparison between young adult and other population eveningness preference to reduce the confounding that is heavily implicit to a priori trait association and suggestibility.

**Thank you for raising this. We have added some additional detail and discussion on these points in the 2^nd^ paragraph of the Introduction, and also in the Discussion – please see final sentence of the Limitations section.**

**References 13, and 66 have additionally been added.**

“and mindfulness-based interventions have been shown to be effective in treating psychological disorders [28–30].” Please unpack this background evidence specifically for depression to ensure consistency from background formulation of evidence for the hypothesis to the investigated variables and even future directions and to not unnecessarily confound forms of symptomatic expression.

**Thank you for this. We have now added some detail and specified the mechanisms implicated.**

**References 30, 31,32, 33, 34, 35 have been added.**

“Therefore, understanding the relationship between alcohol consumption, chronotype, and depression is imperative, but remains understudied.” I am not sure if this understanding is imperative unless the relationship between young adults and alcohol consumption is confirmed.

**The preceding sentences set out the evidence linking chronotype to alcohol use, and how alcohol use impacts mental health- we have rephrased for clarity, but here we are making the point that alcohol could serve as a mediator in the relationship, as rationale for the current work.**

“serving as a likely pathway in the relationship between eveningness and depression” I am not sure how alcohol consumption fits into this pathway although it necessarily does if the effect of alcohol consumption on sleep quality is neither presented nor available, this may be a crucial third variable that is missing.

**We were just reiterating the point made in the previous paragraph regarding the Van den Berg study and alcohol use, but we have removed this now to avoid any confusion.**

“comprehensively identified” Is this an aim of the study, to create scientific consensus that is comprehensive based on identities? If so this would be a non sequitur and I would ask that the authors redact or alter this phrasing seeing as a scientific hypothesis should not be overextended.

**Thank you for this. We have now revised.**

Can the authors explain their reasoning for the use of the reduced version of the rMEQ when additional investigatory depth including statistical rigour is likely needed due to confounding of chronicity and age-related factors in the sampled population? Is the reduced rMEQ statistically equivalent?

**Thank you for raising this. Many studies have** **demonstrated the validity and reliability of the rMEQ, and its equivalence to the MEQ – we now cite 2 relevant studies (reference 58 and 59) in Methods.**

As for the imputed mediating effect of rumination cognitive dys-control just as easily explains the same effect without however positing a model of depression, since the variables included for examination in this study are numerous and heterogenous the authors should be mindful to defer to more general phenomena that may explain likened phenomena.

**Indeed, both rumination and (some facets of) mindfulness can be characterised as elements that relate to ‘cognitive dyscontrol’. Cognitive control deficits are of course importantly related to features of depression, including the actual cognitive deficits such as concentration and memory problems. However, the current study did not focus on cognitive control, or cognitive performance, but we agree these would be interesting areas for future research.**

Citation 64 does not directly identify an effect of rumination on sleep in young adults, in fact the study in citation 64 explores reasons why rumination may not in fact be the single perpetuating factor in terms of the impacted sleep quality of adolescents with depression, rather various heteregenous forms of sleep disturbance, moreover the population in the study in citation 64 is not a homogenous young adult population.

**Thank you for raising this issue. We have now changed that reference to another study which found rumination to mediate the association between chronotype and lifetime depression history in an adult population.**

**Reference 71 has been added.**
