# Supplementary material for: Mindfulness mediates the association between chronotype and depressive symptoms in young adults
Source: PLoS One. 2025 Mar 19;20(3):e0319915. doi: 10.1371/journal.pone.0319915 (PMC11922220; doi:10.1371/journal.pone.0319915)
Supplement: Peer review history

# Author comment: Author response to Decision Letter 0

**DOI:** 10.1371/journal.pone.0319915.r001

17 May 2024

# Peer review: Decision Letter 0

**DOI:** 10.1371/journal.pone.0319915.r002

**Authors:** Beata Peplonska

16 Jul 2024

PONE-D-24-14793Mindfulness mediates the association between chronotype and depressive symptoms in young adultsPLOS ONE

Dear Dr. Yatagan Sevim,

Thank you for submitting your manuscript to PLOS ONE. After careful consideration, we feel that it has merit but does not fully meet PLOS ONE’s publication criteria as it currently stands. Therefore, we invite you to submit a revised version of the manuscript that addresses the points raised during the review process.

Please submit your revised manuscript by Aug 30 2024 11:59PM. If you will need more time than this to complete your revisions, please reply to this message or contact the journal office at plosone@plos.org . When you're ready to submit your revision, log on to https://www.editorialmanager.com/pone/ and select the 'Submissions Needing Revision' folder to locate your manuscript file.

Please include the following items when submitting your revised manuscript:

- A rebuttal letter that responds to each point raised by the academic editor and reviewer(s). You should upload this letter as a separate file labeled 'Response to Reviewers'.
- A marked-up copy of your manuscript that highlights changes made to the original version. You should upload this as a separate file labeled 'Revised Manuscript with Track Changes'.
- An unmarked version of your revised paper without tracked changes. You should upload this as a separate file labeled 'Manuscript'.

If you would like to make changes to your financial disclosure, please include your updated statement in your cover letter. Guidelines for resubmitting your figure files are available below the reviewer comments at the end of this letter.

If applicable, we recommend that you deposit your laboratory protocols in protocols.io to enhance the reproducibility of your results. Protocols.io assigns your protocol its own identifier (DOI) so that it can be cited independently in the future. For instructions see: https://journals.plos.org/plosone/s/submission-guidelines#loc-laboratory-protocols . Additionally, PLOS ONE offers an option for publishing peer-reviewed Lab Protocol articles, which describe protocols hosted on protocols.io. Read more information on sharing protocols at https://plos.org/protocols?utm_medium=editorial-email&utm_source=authorletters&utm_campaign=protocols .

We look forward to receiving your revised manuscript.

Kind regards,

Beata Peplonska

Academic Editor

PLOS ONE

Journal Requirements:

When submitting your revision, we need you to address these additional requirements.

1. Please ensure that your manuscript meets PLOS ONE's style requirements, including those for file naming. The PLOS ONE style templates can be found at 

https://journals.plos.org/plosone/s/file?id=wjVg/PLOSOne_formatting_sample_main_body.pdf and 

https://journals.plos.org/plosone/s/file?id=ba62/PLOSOne_formatting_sample_title_authors_affiliations.pdf

2. We note that your Data Availability Statement is currently as follows: [All relevant data are within the manuscript and its Supporting Information files.]

Please confirm at this time whether or not your submission contains all raw data required to replicate the results of your study. Authors must share the “minimal data set” for their submission. PLOS defines the minimal data set to consist of the data required to replicate all study findings reported in the article, as well as related metadata and methods (https://journals.plos.org/plosone/s/data-availability#loc-minimal-data-set-definition).

For example, authors should submit the following data:

- The values behind the means, standard deviations and other measures reported;

- The values used to build graphs;

- The points extracted from images for analysis.

Authors do not need to submit their entire data set if only a portion of the data was used in the reported study.

If your submission does not contain these data, please either upload them as Supporting Information files or deposit them to a stable, public repository and provide us with the relevant URLs, DOIs, or accession numbers. For a list of recommended repositories, please see https://journals.plos.org/plosone/s/recommended-repositories.

If there are ethical or legal restrictions on sharing a de-identified data set, please explain them in detail (e.g., data contain potentially sensitive information, data are owned by a third-party organization, etc.) and who has imposed them (e.g., an ethics committee). Please also provide contact information for a data access committee, ethics committee, or other institutional body to which data requests may be sent. If data are owned by a third party, please indicate how others may request data access.

3. Please include captions for your Supporting Information files at the end of your manuscript, and update any in-text citations to match accordingly. Please see our Supporting Information guidelines for more information: http://journals.plos.org/plosone/s/supporting-information. 

Additional Editor Comments:

The study methods description should be better elaborated. In specific, information about study population, incl. source, target population; eligibility, inclusion and exclusion criteria. How the eligible subjects were invited, how many were eligible and how many participated (% of the eligible). Was the selection bias excluded? The limitation described in the discussion should consider also potential for selection bias and whether it was excluded.

[Note: HTML markup is below. Please do not edit.]

Reviewers' comments:

Reviewer's Responses to Questions

**Comments to the Author**

1. Is the manuscript technically sound, and do the data support the conclusions?

The manuscript must describe a technically sound piece of scientific research with data that supports the conclusions. Experiments must have been conducted rigorously, with appropriate controls, replication, and sample sizes. The conclusions must be drawn appropriately based on the data presented. 

Reviewer #1: Partly

Reviewer #2: Partly

**********

2. Has the statistical analysis been performed appropriately and rigorously? 

Reviewer #1: Yes

Reviewer #2: Yes

**********

3. Have the authors made all data underlying the findings in their manuscript fully available?

The PLOS Data policy requires authors to make all data underlying the findings described in their manuscript fully available without restriction, with rare exception (please refer to the Data Availability Statement in the manuscript PDF file). The data should be provided as part of the manuscript or its supporting information, or deposited to a public repository. For example, in addition to summary statistics, the data points behind means, medians and variance measures should be available. If there are restrictions on publicly sharing data—e.g. participant privacy or use of data from a third party—those must be specified.

Reviewer #1: Yes

Reviewer #2: No

**********

4. Is the manuscript presented in an intelligible fashion and written in standard English?

PLOS ONE does not copyedit accepted manuscripts, so the language in submitted articles must be clear, correct, and unambiguous. Any typographical or grammatical errors should be corrected at revision, so please note any specific errors here.

Reviewer #1: Yes

Reviewer #2: No

**********

5. Review Comments to the Author

Please use the space provided to explain your answers to the questions above. You may also include additional comments for the author, including concerns about dual publication, research ethics, or publication ethics. (Please upload your review as an attachment if it exceeds 20,000 characters)

Reviewer #1: My main concern about the article is that a large number of potential mediators was considered. Thus, the aim and hypothesis is quite scattered into different areas, i.e. psychological traits (mindfulness, tendency to rumination), sleep quality and lifestyle factors (alcohol consumption), but those variables are treated equally. Also, as state, a parallel mediation analysis was conducted - as I understand, separate models were constructed for each potential mediator. Please qualify.

I suggest to reduce the number of potential variables of interest (mediators) and run a simultaneous, not parallel mediation analysis, to include all the potential mediatiors in one model and thus to better elucidate which factors exactly are responsible for the total mediation between chronotype and depressive symptoms. I believe the sample size is enough to run such an analysis, especially that the bootstrapping is stated to be utilized. This would improve the quality of this research. The discussion should be updated accordingly then.

Minor issues:

Table 2 is a bit hard to read - I suggest removing the exact p values and marking the significant correlations with symbols for thresholds p<0.05, p<0.01 and p<0.001.

The utilized abbreviations are not explained in the Tables.

I am not sure if marking the indirect effect of IV on DV with “a x b” is correct, because this denotations suggests a moderation (or interaction), not mediation. Please verify this.

What is the measure of the Effect in the mediation analysis? Is it a standardised or non-standardises parameter? Please clarify, I suggest presenting the standardised parameters to enable interpratation in terms of effect size.

I also have doubt whether the table contains the parameters resulted from bootstrapping - usually Bootstrap provides effects with their effect sizes, but not p values. Consequently, the statistical significance of the associations is assessed by confidence intervals, not p values. Please verify this and explain.

Reviewer #2: 1. While analyzing association between chronotype and depressive symptoms is a worthwhile topic, my biggest concern refers to mechanism that the Authors claim to have been proven by previous and the current research. Overall, I disagree with the causality imposed by the Authors in multiple places, from title to discussion. The are no proofs that evening chronotype leads to depression and the opposite direction is similarly likely. Therefore, I urge the Authors to be more caution in these statements and modify them so that researchers’ intepretations are separated from studies results. The cross-sectional desing of this study excludes possibility to analyze true mediation [as opposed to statistical – in fact confounder analysis in this study], and is even more undermined by the Authors’ plan to “explore” [mediation in cross-sectional data can be inferred only if there is a strong background proving causality in links between the studied variables, e.g., chronotype->mindfulness). So, I urge tha Authors to remove all statements imposing causality, stating this is a confounder analysis, while causality mechanism can be suggested as a part of introduction/discussion, but not as a proved fact, bur rather a speculation.

2. Division into chronotype groups is incorrect – the Authors analyze continuous variable and it should be analyzed in its full spectrum instead of creating artifical groups based on arbitrary derived cutoff values that result great size inbalance of the groups.

3. The Authors only briefly mention sex differences in chronotype. They need to discuss them in more detail, indicating that they are well-documented [https://doi.org/10.1080/07420528.2019.1585867] and have biological underpinnings [https://doi.org/10.1016/j.psyneuen.2019.05.027].

4. The term “circadian preference” semantically is incorrect, please use morningness-eveningness or chronotype or time-of-day preference.

5. It is odd that the Authors analyzed planned contrasts for exploaratory purposes without hypotheses. They should rather apply bonferroni correction for multiple comparisons.

6. When writing about negative bias in chronotypes no reference is enclosed. Please provide a reference [https://doi.org/10.1007/s11920-018-0925-8]

7. Please be consistent in using sex/gender [only one of this – sex I believe is more valid here]

8. Proofreading by a native English speaker is advised [e.g., “an morningness”]

**********

6. PLOS authors have the option to publish the peer review history of their article (what does this mean? ). If published, this will include your full peer review and any attached files.

If you choose “no”, your identity will remain anonymous but your review may still be made public.

**Do you want your identity to be public for this peer review?** For information about this choice, including consent withdrawal, please see our Privacy Policy .

Reviewer #1: **Yes: ** Lukasz Mokros

Reviewer #2: No

**********

[NOTE: If reviewer comments were submitted as an attachment file, they will be attached to this email and accessible via the submission site. Please log into your account, locate the manuscript record, and check for the action link "View Attachments". If this link does not appear, there are no attachment files.]

While revising your submission, please upload your figure files to the Preflight Analysis and Conversion Engine (PACE) digital diagnostic tool, https://pacev2.apexcovantage.com/ . PACE helps ensure that figures meet PLOS requirements. To use PACE, you must first register as a user. Registration is free. Then, login and navigate to the UPLOAD tab, where you will find detailed instructions on how to use the tool. If you encounter any issues or have any questions when using PACE, please email PLOS at figures@plos.org . Please note that Supporting Information files do not need this step.

# Author comment: Author response to Decision Letter 1

**DOI:** 10.1371/journal.pone.0319915.r003

25 Jul 2024

Additional Editor Comments:

The study methods description should be better elaborated. In specific, information about study population, incl. source, target population; eligibility, inclusion and exclusion criteria. How the eligible subjects were invited, how many were eligible and how many participated (% of the eligible). Was the selection bias excluded? The limitation described in the discussion should consider also potential for selection bias and whether it was excluded.

Thank you for this- we have added detail in the Methods, and in the Limitations.

Reviewers' comments:

Reviewer's Responses to Questions

Comments to the Author

1. Is the manuscript technically sound, and do the data support the conclusions?

The manuscript must describe a technically sound piece of scientific research with data that supports the conclusions. Experiments must have been conducted rigorously, with appropriate controls, replication, and sample sizes. The conclusions must be drawn appropriately based on the data presented.

Reviewer #1: Partly

Reviewer #2: Partly

________________________________________

2. Has the statistical analysis been performed appropriately and rigorously?

Reviewer #1: Yes

Reviewer #2: Yes

________________________________________

3. Have the authors made all data underlying the findings in their manuscript fully available?

The PLOS Data policy requires authors to make all data underlying the findings described in their manuscript fully available without restriction, with rare exception (please refer to the Data Availability Statement in the manuscript PDF file). The data should be provided as part of the manuscript or its supporting information, or deposited to a public repository. For example, in addition to summary statistics, the data points behind means, medians and variance measures should be available. If there are restrictions on publicly sharing data—e.g. participant privacy or use of data from a third party—those must be specified.

Reviewer #1: Yes

Reviewer #2: No

________________________________________

4. Is the manuscript presented in an intelligible fashion and written in standard English?

PLOS ONE does not copyedit accepted manuscripts, so the language in submitted articles must be clear, correct, and unambiguous. Any typographical or grammatical errors should be corrected at revision, so please note any specific errors here.

Reviewer #1: Yes

Reviewer #2: No

________________________________________

5. Review Comments to the Author

Please use the space provided to explain your answers to the questions above. You may also include additional comments for the author, including concerns about dual publication, research ethics, or publication ethics. (Please upload your review as an attachment if it exceeds 20,000 characters)

Reviewer #1: My main concern about the article is that a large number of potential mediators was considered. Thus, the aim and hypothesis is quite scattered into different areas, i.e. psychological traits (mindfulness, tendency to rumination), sleep quality and lifestyle factors (alcohol consumption), but those variables are treated equally. Also, as state, a parallel mediation analysis was conducted - as I understand, separate models were constructed for each potential mediator. Please qualify.

I suggest to reduce the number of potential variables of interest (mediators) and run a simultaneous, not parallel mediation analysis, to include all the potential mediatiors in one model and thus to better elucidate which factors exactly are responsible for the total mediation between chronotype and depressive symptoms. I believe the sample size is enough to run such an analysis, especially that the bootstrapping is stated to be utilized. This would improve the quality of this research. The discussion should be updated accordingly then.

Thank you for raising this and apologies for any lack of clarity. The mediating roles of variables were assessed simultaneously using a single parallel mediation model (Hayes Model 4) by including all variables as mediators in the model. We have now made this clearer throughout that the multiple mediators were assessed simultaneously.

Minor issues:

Table 2 is a bit hard to read - I suggest removing the exact p values and marking the significant correlations with symbols for thresholds p<0.05, p<0.01 and p<0.001.

Thank you. We have now removed p-values from table and used symbols to indicate significance.

The utilized abbreviations are not explained in the Tables.

Thank you, we have now added the explanation for abbreviations used in the tables as a note below the table where necessary.

I am not sure if marking the indirect effect of IV on DV with „a x b” is correct, because this denotations suggests a moderation (or interaction), not mediation. Please verify this.

Thank you for the comment. We have now changed to this to “ab” to represent the indirect effect.

What is the measure of the Effect in the mediation analysis? Is it a standardised or non-standardises parameter? Please clarify, I suggest presenting the standardised parameters to enable interpratation in terms of effect size.

Thank you for raising this. We have now made clear in the method section that all are standardized coefficients.

I also have doubt whether the table contains the parameters resulted from bootstrapping - usually Bootstrap provides effects with their effect sizes, but not p values. Consequently, the statistical significance of the associations is assessed by confidence intervals, not p values. Please verify this and explain.

Thank you for this comment, we report the bootstrap confidence intervals for all indirect effects, and this is now specified in the method and results sections.

Reviewer #2: 1. While analyzing association between chronotype and depressive symptoms is a worthwhile topic, my biggest concern refers to mechanism that the Authors claim to have been proven by previous and the current research. Overall, I disagree with the causality imposed by the Authors in multiple places, from title to discussion. The are no proofs that evening chronotype leads to depression and the opposite direction is similarly likely. Therefore, I urge the Authors to be more caution in these statements and modify them so that researchers’ intepretations are separated from studies results. The cross-sectional desing of this study excludes possibility to analyze true mediation [as opposed to statistical – in fact confounder analysis in this study], and is even more undermined by the Authors’ plan to “explore” [mediation in cross-sectional data can be inferred only if there is a strong background proving causality in links between the studied variables, e.g., chronotype->mindfulness). So, I urge tha Authors to remove all statements imposing causality, stating this is a confounder analysis, while causality mechanism can be suggested as a part of introduction/discussion, but not as a proved fact, bur rather a speculation.

We thank the reviewer for raising this important point. For brevity, the introduction did not fully set out the evidence supporting a causal relationship between evening chronotype and depression and we apologise for the omission, this is of course essential given that the mediation analysis is predicated upon it. There is considerable evidence showing higher risk amongst evening types, for example:

Kivelä, L., Papadopoulos, M. R. & Antypa, N. Chronotype and psychiatric disorders. Curr. Sleep Med. Rep. https://doi.org/10.1007/s40675-018-0113-8 (2018).

Taylor, B. J. & Hasler, B. P. Chronotype and mental health: Recent advances. Curr. Psychiatry Rep. 20, 59 (2018).

Kitamura, S. et al. Evening preference is related to the incidence of depressive states independent of sleep-wake conditions. Chronobiol. Int. 27, 1797–1812 (2010).

Merikanto, I. et al. Evening types are prone to depression. Chronobiol. Int. 30, 719–725 (2013).

Hidalgo, M. P. et al. Relationship between depressive mood and chronotype in healthy subjects. Psychiatry Clin. Neurosci. 63, 283–290 (2009).

Antypa, N., Vogelzangs, N., Meesters, Y., Schoevers, R. & Penninx, B. W. J. H. Chronotype associations with depression and anxiety disorders in a large cohort study. Depress. Anxiety https://doi.org/10.1002/da.22422 (2015).

Merikanto, I. et al. Circadian preference links to depression in general adult population. J. Affect. Disord. 188, 143–148 (2015).

Chan, J. W. Y. et al. Eveningness and insomnia: Independent Risk factors of nonremission in major depressive disorder. Sleep https://doi.org/10.5665/sleep.3658 (2014).

Gaspar-Barba, E. et al. Depressive symptomatology is influenced by chronotypes. J. Affect. Disord. 119, 100–106 (2009).

Bauducco, S., Richardson, C. & Gradisar, M. Chronotype, circadian rhythms and mood. Curr. Opin. Psychol. 34, 77–83 (2020).

Taillard, J., Sagaspe, P., Philip, P. & Bioulac, S. Sleep timing, chronotype and social jetlag: Impact on cognitive abilities and psychiatric disorders. Biochem. Pharmacol. 1, 114438. https://doi.org/10.1016/j.bcp.2021.114438 (2021).

While there is no doubt that depression is associated with sleep disturbance (and likely bidirectional for that relationship), we are not aware of any studies suggesting that depression can itself affect chronotype. Further, it should be mentioned that diurnal preference is considered a relatively stable trait in adulthood. Twin studies indicate heritability estimates between 46 and 57% for diurnal preference (Barclay, N. L., Watson, N. F., Buchwald, D. & Goldberg, J. Moderation of genetic and environmental influences on diurnal preference by age in adult twins. Chronobiol. Int. 31, 222–231 (2014); Barclay, N. L., Rowe, R., O’Leary, R., Bream, D. & Gregory, A. M. Longitudinal stability of genetic and environmental influences on the association between diurnal preference and sleep quality in young adult twins and siblings. J. Biol. Rhythms 31, 375–386 (2016)). Given its trait-like characteristics, this further supports treating chronotype as the causal factor in the relationship, rather than depression.

We have expanded the Introduction to justify our approach and we hope that this now makes the rationale clearer.

Line 72:

In general, there is a large body of work showing an association between eveningness and both subclinical and clinical depression symptoms (16). A meta-analysis (n =15734) found consistent evidence that an eveningness orientation is associated with a higher risk of depressive symptoms (5); only one of the 36 studies included failed to find a relationship.

2. Division into chronotype groups is incorrect – the Authors analyze continuous variable and it should be analyzed in its full spectrum instead of creating artifical groups based on arbitrary derived cutoff values that result great size inbalance of the groups.

For the first hypothesis, we compare between chronotype groups, but this is based on the well-established (and well-used) cut-offs for the MEQ (not arbitrary/derived values): these cut-offs have been externally validated against circadian motor activity. We now add more detail in the Measures section. For the mediation analysis, this is based on the continuous variable; again we now make this clearer throughout.

3. The Authors only briefly mention sex differences in chronotype. They need to discuss them in more detail, indicating that they are well-documented [https://doi.org/10.1080/07420528.2019.1585867] and have biological underpinnings [https://doi.org/10.1016/j.psyneuen.2019.05.027].

Thank you. We have now expanded on sex differences in chronotype, and added these references, in discussion (line 389)

4. The term “circadian preference” semantically is incorrect, please use morningness-eveningness or chronotype or time-of-day preference.

Thank you. We have now changed “circadian preference” to “morningness-eveningness” or “chronotype” throughout the manuscript.

5. It is odd that the Authors analyzed planned contrasts for exploaratory purposes without hypotheses. They should rather apply bonferroni correction for multiple comparisons.

Thank you for this. In hypothesis 1, we specifically hypothesised “Compared to morning and intermediate types, evening types will show significantly higher levels of depression, rumination, and alcohol consumption, lower levels of mindfulness, and poorer sleep quality”, thus, the analysis approach we use (planned contrasts with Bonferroni correction) is appropriate, please see Methods.

6. When writing about negative bias in chronotypes no reference is enclosed. Please provide a reference [https://doi.org/10.1007/s11920-018-0925-8]

Thank you for raising this. We have now added this reference as well as one other study which shows negative bias in late chronotype compared to intermediate and morning chronotypes (line 61).

7. Please be consistent in using sex/gender [only one of this – sex I believe is more valid here]

Thank you. We have now changed “gender” to “sex” where we discuss our sample/results.

8. Proofreading by a native English speaker is advised [e.g., “an morningness”]

Thank you for this. We have checked through and corrected.

Attachment Submitted filename: Response to Reviewers.docx

# Peer review: Decision Letter 1

**DOI:** 10.1371/journal.pone.0319915.r004

**Authors:** Beata Peplonska

16 Dec 2024

PONE-D-24-14793R1Mindfulness mediates the association between chronotype and depressive symptoms in young adultsPLOS ONE

Dear Dr. Yatagan Sevim,

Thank you for submitting your manuscript to PLOS ONE. After careful consideration, we feel that it has merit but does not fully meet PLOS ONE’s publication criteria as it currently stands. Therefore, we invite you to submit a revised version of the manuscript that addresses the points raised during the review process.

The additional evaluation for the revied version has been obtained. Please, address issues raised by the Reviewer.   

Please submit your revised manuscript by Jan 30 2025 11:59PM. If you will need more time than this to complete your revisions, please reply to this message or contact the journal office at plosone@plos.org . When you're ready to submit your revision, log on to https://www.editorialmanager.com/pone/ and select the 'Submissions Needing Revision' folder to locate your manuscript file.

Please include the following items when submitting your revised manuscript:

- A rebuttal letter that responds to each point raised by the academic editor and reviewer(s). You should upload this letter as a separate file labeled 'Response to Reviewers'.
- A marked-up copy of your manuscript that highlights changes made to the original version. You should upload this as a separate file labeled 'Revised Manuscript with Track Changes'.
- An unmarked version of your revised paper without tracked changes. You should upload this as a separate file labeled 'Manuscript'.

If you would like to make changes to your financial disclosure, please include your updated statement in your cover letter. Guidelines for resubmitting your figure files are available below the reviewer comments at the end of this letter.

If applicable, we recommend that you deposit your laboratory protocols in protocols.io to enhance the reproducibility of your results. Protocols.io assigns your protocol its own identifier (DOI) so that it can be cited independently in the future. For instructions see: https://journals.plos.org/plosone/s/submission-guidelines#loc-laboratory-protocols . Additionally, PLOS ONE offers an option for publishing peer-reviewed Lab Protocol articles, which describe protocols hosted on protocols.io. Read more information on sharing protocols at https://plos.org/protocols?utm_medium=editorial-email&utm_source=authorletters&utm_campaign=protocols .

We look forward to receiving your revised manuscript.

Kind regards,

Beata Peplonska

Academic Editor

PLOS ONE

Journal Requirements:

Please review your reference list to ensure that it is complete and correct. If you have cited papers that have been retracted, please include the rationale for doing so in the manuscript text, or remove these references and replace them with relevant current references. Any changes to the reference list should be mentioned in the rebuttal letter that accompanies your revised manuscript. If you need to cite a retracted article, indicate the article’s retracted status in the References list and also include a citation and full reference for the retraction notice.

[Note: HTML markup is below. Please do not edit.]

Reviewers' comments:

Reviewer's Responses to Questions

**Comments to the Author**

1. If the authors have adequately addressed your comments raised in a previous round of review and you feel that this manuscript is now acceptable for publication, you may indicate that here to bypass the “Comments to the Author” section, enter your conflict of interest statement in the “Confidential to Editor” section, and submit your "Accept" recommendation.

Reviewer #2: All comments have been addressed

Reviewer #3: All comments have been addressed

**********

2. Is the manuscript technically sound, and do the data support the conclusions?

The manuscript must describe a technically sound piece of scientific research with data that supports the conclusions. Experiments must have been conducted rigorously, with appropriate controls, replication, and sample sizes. The conclusions must be drawn appropriately based on the data presented. 

Reviewer #2: Yes

Reviewer #3: Yes

**********

3. Has the statistical analysis been performed appropriately and rigorously? 

Reviewer #2: Yes

Reviewer #3: Yes

**********

4. Have the authors made all data underlying the findings in their manuscript fully available?

The PLOS Data policy requires authors to make all data underlying the findings described in their manuscript fully available without restriction, with rare exception (please refer to the Data Availability Statement in the manuscript PDF file). The data should be provided as part of the manuscript or its supporting information, or deposited to a public repository. For example, in addition to summary statistics, the data points behind means, medians and variance measures should be available. If there are restrictions on publicly sharing data—e.g. participant privacy or use of data from a third party—those must be specified.

Reviewer #2: Yes

Reviewer #3: Yes

**********

5. Is the manuscript presented in an intelligible fashion and written in standard English?

PLOS ONE does not copyedit accepted manuscripts, so the language in submitted articles must be clear, correct, and unambiguous. Any typographical or grammatical errors should be corrected at revision, so please note any specific errors here.

Reviewer #2: Yes

Reviewer #3: Yes

**********

6. Review Comments to the Author

Please use the space provided to explain your answers to the questions above. You may also include additional comments for the author, including concerns about dual publication, research ethics, or publication ethics. (Please upload your review as an attachment if it exceeds 20,000 characters)

Reviewer #2: Thanks for your work. All comments I raised in my review have been adressed by the Authors adequately.

Reviewer #3: This scholarly article is well-written, written in plain language, well-structured and its analyses are sound, however I do have critical feedback to help inform potential publication at the current stage in the review process.

“a large proportion of individuals aged 17 to 25 are typically categorised as evening-types” The authors ought to directly evaluate the validity of their findings based on this item of demographic information in the form of a statement of limitation, is the effect being examined a potential function of observer bias, p-fishing or a direct contribution from another extraneous, likely neurophysiological factor such as hormone uptake, eye movements and photon displacement, etc., more suitable for experimental analysis and scientific contribution based on the phenomenon in question? Eveningness preference especially is heavily empirically and ex post facto a less mindful choice than any other form of chronicity, thus potentially disrupting the reliability of the reporting, even if it is explored later in the background evidence in terms of mindfulness chronicity is not a priori a direct function of mindfulness but of biology.

As another reviewer commented in a previous review there is indeed a potentially exaggerated extent of causal inference being made in this paper in particular about the mediating properties of the phenomenon of diurnal preference reported to be endemic, not abnormal, in its sampled population. At the very least I would expect some background comparison between young adult and other population eveningness preference to reduce the confounding that is heavily implicit to a priori trait association and suggestibility.

“and mindfulness-based interventions have been shown to be effective in treating psychological disorders [28–30].” Please unpack this background evidence specifically for depression to ensure consistency from background formulation of evidence for the hypothesis to the investigated variables and even future directions and to not unnecessarily confound forms of symptomatic expression.

“Therefore, understanding the relationship between alcohol consumption, chronotype, and depression is imperative, but remains understudied.” I am not sure if this understanding is imperative unless the relationship between young adults and alcohol consumption is confirmed.

“serving as a likely pathway in the relationship between eveningness and depression” I am not sure how alcohol consumption fits into this pathway although it necessarily does if the effect of alcohol consumption on sleep quality is neither presented nor available, this may be a crucial third variable that is missing.

“comprehensively identified” Is this an aim of the study, to create scientific consensus that is comprehensive based on identities? If so this would be a non sequitur and I would ask that the authors redact or alter this phrasing seeing as a scientific hypothesis should not be overextended.

Can the authors explain their reasoning for the use of the reduced version of the rMEQ when additional investigatory depth including statistical rigour is likely needed due to confounding of chronicity and age-related factors in the sampled population? Is the reduced rMEQ statistically equivalent?

As for the imputed mediating effect of rumination cognitive dys-control just as easily explains the same effect without however positing a model of depression, since the variables included for examination in this study are numerous and heterogenous the authors should be mindful to defer to more general phenomena that may explain likened phenomena.

Citation 64 does not directly identify an effect of rumination on sleep in young adults, in fact the study in citation 64 explores reasons why rumination may not in fact be the single perpetuating factor in terms of the impacted sleep quality of adolescents with depression, rather various heteregenous forms of sleep disturbance, moreover the population in the study in citation 64 is not a homogenous young adult population.

**********

7. PLOS authors have the option to publish the peer review history of their article (what does this mean? ). If published, this will include your full peer review and any attached files.

If you choose “no”, your identity will remain anonymous but your review may still be made public.

**Do you want your identity to be public for this peer review?** For information about this choice, including consent withdrawal, please see our Privacy Policy .

Reviewer #2: No

Reviewer #3: **Yes: ** Paul-André Betito, HBA*, MSW, RSW

**********

[NOTE: If reviewer comments were submitted as an attachment file, they will be attached to this email and accessible via the submission site. Please log into your account, locate the manuscript record, and check for the action link "View Attachments". If this link does not appear, there are no attachment files.]

While revising your submission, please upload your figure files to the Preflight Analysis and Conversion Engine (PACE) digital diagnostic tool, https://pacev2.apexcovantage.com/ . PACE helps ensure that figures meet PLOS requirements. To use PACE, you must first register as a user. Registration is free. Then, login and navigate to the UPLOAD tab, where you will find detailed instructions on how to use the tool. If you encounter any issues or have any questions when using PACE, please email PLOS at figures@plos.org . Please note that Supporting Information files do not need this step.

# Author comment: Author response to Decision Letter 2

**DOI:** 10.1371/journal.pone.0319915.r005

20 Jan 2025

Reviewers' comments:

Reviewer's Responses to Questions

Comments to the Author

1. If the authors have adequately addressed your comments raised in a previous round of review and you feel that this manuscript is now acceptable for publication, you may indicate that here to bypass the “Comments to the Author” section, enter your conflict of interest statement in the “Confidential to Editor” section, and submit your "Accept" recommendation.

Reviewer #2: All comments have been addressed

Reviewer #3: All comments have been addressed

________________________________________

2. Is the manuscript technically sound, and do the data support the conclusions?

The manuscript must describe a technically sound piece of scientific research with data that supports the conclusions. Experiments must have been conducted rigorously, with appropriate controls, replication, and sample sizes. The conclusions must be drawn appropriately based on the data presented.

Reviewer #2: Yes

Reviewer #3: Yes

________________________________________

3. Has the statistical analysis been performed appropriately and rigorously?

Reviewer #2: Yes

Reviewer #3: Yes

________________________________________

4. Have the authors made all data underlying the findings in their manuscript fully available?

The PLOS Data policy requires authors to make all data underlying the findings described in their manuscript fully available without restriction, with rare exception (please refer to the Data Availability Statement in the manuscript PDF file). The data should be provided as part of the manuscript or its supporting information, or deposited to a public repository. For example, in addition to summary statistics, the data points behind means, medians and variance measures should be available. If there are restrictions on publicly sharing data—e.g. participant privacy or use of data from a third party—those must be specified.

Reviewer #2: Yes

Reviewer #3: Yes

________________________________________

5. Is the manuscript presented in an intelligible fashion and written in standard English?

PLOS ONE does not copyedit accepted manuscripts, so the language in submitted articles must be clear, correct, and unambiguous. Any typographical or grammatical errors should be corrected at revision, so please note any specific errors here.

Reviewer #2: Yes

Reviewer #3: Yes

________________________________________

6. Review Comments to the Author

Please use the space provided to explain your answers to the questions above. You may also include additional comments for the author, including concerns about dual publication, research ethics, or publication ethics. (Please upload your review as an attachment if it exceeds 20,000 characters)

Reviewer #2: Thanks for your work. All comments I raised in my review have been adressed by the Authors adequately.

Reviewer #3: This scholarly article is well-written, written in plain language, well-structured and its analyses are sound, however I do have critical feedback to help inform potential publication at the current stage in the review process.

We thank the reviewer for their positive comments and helpful feedback.

“a large proportion of individuals aged 17 to 25 are typically categorised as evening-types” The authors ought to directly evaluate the validity of their findings based on this item of demographic information in the form of a statement of limitation, is the effect being examined a potential function of observer bias, p-fishing or a direct contribution from another extraneous, likely neurophysiological factor such as hormone uptake, eye movements and photon displacement, etc., more suitable for experimental analysis and scientific contribution based on the phenomenon in question? Eveningness preference especially is heavily empirically and ex post facto a less mindful choice than any other form of chronicity, thus potentially disrupting the reliability of the reporting, even if it is explored later in the background evidence in terms of mindfulness chronicity is not a priori a direct function of mindfulness but of biology.

Indeed, chronicity is largely biologically determined. Apologies if this is not clear, but we are not at all suggesting that mindfulness impacts chronicity – we present mindfulness as an independent factor that could explain the relationship between evening chronicity and poorer mental health. We have added extra detail at the start of the 2nd paragraph emphasise the biological contribution to chronicity.

Reference 10 has been added.

As another reviewer commented in a previous review there is indeed a potentially exaggerated extent of causal inference being made in this paper in particular about the mediating properties of the phenomenon of diurnal preference reported to be endemic, not abnormal, in its sampled population. At the very least I would expect some background comparison between young adult and other population eveningness preference to reduce the confounding that is heavily implicit to a priori trait association and suggestibility.

Thank you for raising this. We have added some additional detail and discussion on these points in the 2nd paragraph of the Introduction, and also in the Discussion – please see final sentence of the Limitations section.

References 13, and 66 have additionally been added.

“and mindfulness-based interventions have been shown to be effective in treating psychological disorders [28–30].” Please unpack this background evidence specifically for depression to ensure consistency from background formulation of evidence for the hypothesis to the investigated variables and even future directions and to not unnecessarily confound forms of symptomatic expression.

Thank you for this. We have now added some detail and specified the mechanisms implicated.

References 30, 31,32, 33, 34, 35 have been added.

“Therefore, understanding the relationship between alcohol consumption, chronotype, and depression is imperative, but remains understudied.” I am not sure if this understanding is imperative unless the relationship between young adults and alcohol consumption is confirmed.

The preceding sentences set out the evidence linking chronotype to alcohol use, and how alcohol use impacts mental health- we have rephrased for clarity, but here we are making the point that alcohol could serve as a mediator in the relationship, as rationale for the current work.

“serving as a likely pathway in the relationship between eveningness and depression” I am not sure how alcohol consumption fits into this pathway although it necessarily does if the effect of alcohol consumption on sleep quality is neither presented nor available, this may be a crucial third variable that is missing.

We were just reiterating the point made in the previous paragraph regarding the Van den Berg study and alcohol use, but we have removed this now to avoid any confusion.

“comprehensively identified” Is this an aim of the study, to create scientific consensus that is comprehensive based on identities? If so this would be a non sequitur and I would ask that the authors redact or alter this phrasing seeing as a scientific hypothesis should not be overextended.

Thank you for this. We have now revised.

Can the authors explain their reasoning for the use of the reduced version of the rMEQ when additional investigatory depth including statistical rigour is likely needed due to confounding of chronicity and age-related factors in the sampled population? Is the reduced rMEQ statistically equivalent?

Thank you for raising this. Many studies have demonstrated the validity and reliability of the rMEQ, and its equivalence to the MEQ – we now cite 2 relevant studies (reference 58 and 59) in Methods.

As for the imputed mediating effect of rumination cognitive dys-control just as easily explains the same effect without however positing a model of depression, since the variables included for examination in this study are numerous and heterogenous the authors should be mindful to defer to more general phenomena that may explain likened phenomena.

Indeed, both rumination and (some facets of) mindfulness can be characterised as elements that relate to ‘cognitive dyscontrol’. Cognitive control deficits are of course importantly related to features of depression, including the actual cognitive deficits such as concentration and memory problems. However, the current study did not focus on cognitive control, or cognitive performance, but we agree these would be interesting areas for future research.

Citation 64 does not directly identify an effect of rumination on sleep in young adults, in fact the study in citation 64 explores reasons why rumination may not in fact be the single perpetuating factor in terms of the impacted sleep quality of adolescents with depression, rather various heteregenous forms of sleep disturbance, moreover the population in the study in citation 64 is not a homogenous young adult population.

Thank you for raising this issue. We have now changed that reference to another study which found rumination to mediate the association between chronotype and lifetime depression history in an adult population.

Reference 71 has been added.

Attachment Submitted filename: Response_to_reviewers_auresp_2.docx

# Peer review: Decision Letter 2

**DOI:** 10.1371/journal.pone.0319915.r006

**Authors:** Beata Peplonska

11 Feb 2025

Mindfulness mediates the association between chronotype and depressive symptoms in young adults

PONE-D-24-14793R2

Dear Dr. Yatagan Sevim,

We’re pleased to inform you that your manuscript has been judged scientifically suitable for publication and will be formally accepted for publication once it meets all outstanding technical requirements.

Within one week, you’ll receive an e-mail detailing the required amendments. When these have been addressed, you’ll receive a formal acceptance letter and your manuscript will be scheduled for publication.

An invoice will be generated when your article is formally accepted. Please note, if your institution has a publishing partnership with PLOS and your article meets the relevant criteria, all or part of your publication costs will be covered. Please make sure your user information is up-to-date by logging into Editorial Manager at Editorial Manager®  and clicking the ‘Update My Information' link at the top of the page. If you have any questions relating to publication charges, please contact our Author Billing department directly at authorbilling@plos.org.

If your institution or institutions have a press office, please notify them about your upcoming paper to help maximize its impact. If they’ll be preparing press materials, please inform our press team as soon as possible -- no later than 48 hours after receiving the formal acceptance. Your manuscript will remain under strict press embargo until 2 pm Eastern Time on the date of publication. For more information, please contact onepress@plos.org.

Kind regards,

Beata Peplonska

Academic Editor

PLOS ONE

Additional Editor Comments (optional):

Reviewers' comments:

Reviewer's Responses to Questions

**Comments to the Author**

1. If the authors have adequately addressed your comments raised in a previous round of review and you feel that this manuscript is now acceptable for publication, you may indicate that here to bypass the “Comments to the Author” section, enter your conflict of interest statement in the “Confidential to Editor” section, and submit your "Accept" recommendation.

Reviewer #3: All comments have been addressed

**********

2. Is the manuscript technically sound, and do the data support the conclusions?

The manuscript must describe a technically sound piece of scientific research with data that supports the conclusions. Experiments must have been conducted rigorously, with appropriate controls, replication, and sample sizes. The conclusions must be drawn appropriately based on the data presented. 

Reviewer #3: Yes

**********

3. Has the statistical analysis been performed appropriately and rigorously? 

Reviewer #3: Yes

**********

4. Have the authors made all data underlying the findings in their manuscript fully available?

The PLOS Data policy requires authors to make all data underlying the findings described in their manuscript fully available without restriction, with rare exception (please refer to the Data Availability Statement in the manuscript PDF file). The data should be provided as part of the manuscript or its supporting information, or deposited to a public repository. For example, in addition to summary statistics, the data points behind means, medians and variance measures should be available. If there are restrictions on publicly sharing data—e.g. participant privacy or use of data from a third party—those must be specified.

Reviewer #3: Yes

**********

5. Is the manuscript presented in an intelligible fashion and written in standard English?

PLOS ONE does not copyedit accepted manuscripts, so the language in submitted articles must be clear, correct, and unambiguous. Any typographical or grammatical errors should be corrected at revision, so please note any specific errors here.

Reviewer #3: Yes

**********

6. Review Comments to the Author

Please use the space provided to explain your answers to the questions above. You may also include additional comments for the author, including concerns about dual publication, research ethics, or publication ethics. (Please upload your review as an attachment if it exceeds 20,000 characters)

Reviewer #3: (No Response)

**********

7. PLOS authors have the option to publish the peer review history of their article (what does this mean? ). If published, this will include your full peer review and any attached files.

If you choose “no”, your identity will remain anonymous but your review may still be made public.

**Do you want your identity to be public for this peer review?** For information about this choice, including consent withdrawal, please see our Privacy Policy .

Reviewer #3: **Yes: ** Paul-André Betito, HBA*, MSW, RSW

**********

# Editor report: Acceptance letter

**DOI:** 10.1371/journal.pone.0319915.r007

**Authors:** Beata Peplonska

PONE-D-24-14793R2

PLOS ONE

Dear Dr. Yatagan Sevim,

I'm pleased to inform you that your manuscript has been deemed suitable for publication in PLOS ONE. Congratulations! Your manuscript is now being handed over to our production team.

At this stage, our production department will prepare your paper for publication. This includes ensuring the following:

* All references, tables, and figures are properly cited

* All relevant supporting information is included in the manuscript submission,

* There are no issues that prevent the paper from being properly typeset

If revisions are needed, the production department will contact you directly to resolve them. If no revisions are needed, you will receive an email when the publication date has been set. At this time, we do not offer pre-publication proofs to authors during production of the accepted work. Please keep in mind that we are working through a large volume of accepted articles, so please give us a few weeks to review your paper and let you know the next and final steps.

Lastly, if your institution or institutions have a press office, please let them know about your upcoming paper now to help maximize its impact. If they'll be preparing press materials, please inform our press team within the next 48 hours. Your manuscript will remain under strict press embargo until 2 pm Eastern Time on the date of publication. For more information, please contact onepress@plos.org.

If we can help with anything else, please email us at customercare@plos.org.

Thank you for submitting your work to PLOS ONE and supporting open access.

Kind regards,

PLOS ONE Editorial Office Staff

on behalf of

Dr. Beata Peplonska

Academic Editor

PLOS ONE
